# The downregulated membrane expression of CD18 in CD34^+^ cells defines a primitive population of human hematopoietic stem cells

**DOI:** 10.1186/s13287-020-01672-0

**Published:** 2020-04-28

**Authors:** Cristina Mesa-Núñez, Diego Leon-Rico, Montserrat Aldea, Carlos Damián, Raquel Sanchez-Baltasar, Rebeca Sanchez, Omaira Alberquilla, José Carlos Segovia, Juan Antonio Bueren, Elena Almarza

**Affiliations:** 1 Division of Hematopoietic Innovative Therapies, Centro de Investigaciones Energéticas, Medioambientales y Tecnológicas (CIEMAT), Av. Complutense 40, 28040 Madrid, Spain; 2 Centro de Investigación Biomédica en Red de Enfermedades Raras (CIBERER), Melchor Fernández Almagro 3, 28029 Madrid, Spain; 3 Instituto de Investigación Sanitaria Fundación Jiménez Díaz (IIS- FJD, UAM), Av. de los Reyes Católicos 2, 28040 Madrid, Spain

**Keywords:** Hematopoietic stem cells, CD18, Integrins, Long-term repopulating cells

## Abstract

**Background:**

CD18 is the common beta subunit of β_2_ integrins, which are expressed on hematopoietic cells. β_2_ integrins are essential for cell adhesion and leukocyte trafficking.

**Methods:**

Here we have analyzed the expression of CD18 in different subsets of human hematopoietic stem and progenitor cells (HSPCs) from cord blood (CB), bone marrow (BM), and mobilized peripheral blood (mPB) samples. CD34^+^ cells were classified into CD18^high^ and CD18^low/neg^, and each of these populations was analyzed for the expression of HSPC markers, as well as for their clonogenity, quiescence state, and repopulating ability in immunodeficient mice.

**Results:**

A downregulated membrane expression of CD18 was associated with a primitive hematopoietic stem cells (HSC) phenotype, as well as with a higher content of quiescent cells and multipotent colony-forming cells (CFCs). Although no differences in the short-term repopulating potential of CD18^low/neg^ CD34^+ ^and CD18^high^ CD34^+^ cells were observed, CD18^low/neg^ CD34^+^ cells were characterized by an enhanced long-term repopulating ability in NSG mice.

**Conclusions:**

Overall, our results indicate that the downregulated membrane expression of CD18 characterizes a primitive population of human hematopoietic repopulating cells.

## Significance statement

This study demonstrates that CD18 is a marker whose membrane expression is inversely correlated with the primitiveness of human CD34^+^ cells. The selection of CD34^+^ cells with a reduced expression of CD18 defines a primitive population with an extended repopulating ability as compared with CD34^+^ cells expressing higher levels of CD18.

## Background

Integrins are heterodimeric adhesion molecules that mediate intercellular interactions and cell interactions with the extracellular matrix. β_2_ integrins are composed by a common β_2_ subunit (CD18) and variable α subunits (CD11). The expression of β_2_ integrins is restricted to the hematopoietic system. This integrin subfamily includes four members, whose identity depends on the particular α subunit of the heterodimer. Heterodimers comprised of CD18:CD11A (LFA-1), CD18:CD11B (Mac-1), CD18:CD11C (p150.95), and CD18:CD11D (α_D_β_2_) are associated with distinct patterns of expression and function in the hematopoietic system [1–3]. All leukocytes express the lymphocyte function-associated antigen 1 (LFA-1) on their surface. LFA-1 is mainly involved in the leukocyte adhesion cascade and also in the immune synapse formation between T cells and antigen-presenting cells (APCs). Macrophage 1 antigen (Mac-1) is mostly expressed in the myeloid lineage, where it mediates a range of functions such as phagocytosis, degradation, and remodeling of the extracellular matrix. Integrin p150.95 is expressed in myeloid cells, dendritic cells, NK cells, and populations of activated T and B cells. It mediates phagocytosis of inactive complement component 3b (iC3b)-opsonized particles and it is involved in the adhesion of monocytes to endothelial cells. Integrin α_D_β_2_ is expressed at a basal level on the surface of most leukocytes and is upregulated on phagocytic leukocytes present in regions of local inflammation, although its specific role in leukocyte function remains unknown [3].

Bone marrow HSPCs display a variable expression of LFA-I, although its expression is downregulated by interactions with stromal cells. In the presence of stimulating chemokines, the immune system responds by inducing the proliferation of the HSCs in the BM. In this process, cells which lose stromal interaction will rapidly upregulate LFA-I expression enabling migration to the periphery [4]. Overall, previous reports consistently showed that LFA-I^−^ cells are more pluripotent than LFA-I^+^ cells. Supporting data include a demonstration that LFA-I^−^ cells generate more CFCs in long-term BM cultures than the LFA-I^+^ population. It has been postulated that LFA-I could play a role in the interaction between stromal cells and HSCs, thus allowing HSCs to migrate through the different environments of the stroma [5], but may not be needed for the interaction between the stroma and the most primitive HSCs, implying that LFA-I expression is not an absolute requirement for the earliest stages of hematopoiesis [6]. When studied in mouse models, the transplantation of CD11A^−^ HSCs (defined as Lin^−^c-Kit^+^Sca1^+^CD150^+^CD34^−^) resulted in higher engraftment as compared to similar numbers of CD11A^+^ HSCs, not only in primary but also in secondary recipients. The absence of this marker was also associated with a higher frequency of HSCs in the G_0_ phase of the cell cycle [7], suggesting that the reduced expression of CD11A was associated with primitive HSCs. Similarly, in nonhuman primates, it has been reported that HSCs capable of repopulating both lymphoid and myeloid lineages are also included in the CD34^+^CD11B^−^ fraction [8].

Because the common link between the expression of CD11B and CD11A in the leukocyte membrane is the β subunit (CD18), in the current study, we aimed at providing conclusive evidence regarding the relevance of CD18 expression in human HSPCs. Thus, we investigated the HSPC phenotype and repopulating properties of cord blood, BM, and also mPB CD34^+^ cells either positive or negative for the expression of CD18.

## Methods

### Purification and culture of cord blood CD34^+^ cells

Cord blood samples from healthy donors were obtained from the Madrid Community Transfusion Centre under their Institutional Review Board (IRB) approval and their written informed consent and in compliance with the Helsinki Declaration. Mononuclear cells were isolated by density gradient centrifugation using Ficoll-Paque PLUS (GE Healthcare, Fairfield, USA). These cells were CD34^+^ magnetic-labeled with CD34 MicroBead Kit and separated from the total mononuclear population using LS QuadroMACS™ and MS OctoMACS™ column (MACS, Miltenyi Biotec, Bergisch Gladbach, Germany). Purified CD34^+^ cells were then evaluated for their purity by Flow Cytometry Activated Cell Sorting (FACS), which is regularly around 80–95%.

Freshly isolated CD34^+^ cells were cultured in StemSpan medium (StemCell Technologies, Vancouver, Canada) supplemented with 300 ng/ml hSCF, 100 ng/ml hTPO, 100 ng/ml hFlt3L (Eurobiosciences, Friesoythe, Germany), 1% penicillin/streptomycin (P/S), and 1% GlutaMAX (Gibco/Life Technologies/Thermo Fisher Scientific, Waltham, USA) at 37 °C in 5%CO_2_.

With the aim of characterizing the phenotype and functional properties of CD34^+^ cells based on different levels of CD18 expression, the clone 6.7 of the monoclonal antibody against CD18 was selected to assure recognition of an external domain of the β_2_ chain [9–11] and thus independently on the activation status.

### Flow cytometry analyses and cell sorting

Flow cytometry analyses were performed in the LSRFortessa cell analyzer (BD/Becton, Dickinson and Company, New Jersey, USA). Off-line analyses were made with the FlowJo Software v7.6.5. (Tree star, Ashland, USA).

More than 1 × 10^4^ viable cells were acquired in the LSRFortessa cell analyzer using 1 μg/ml DAPI as a viability marker suspended in flow cytometry buffer consisting of phosphate-buffered saline with 0.5% bovine serum albumin and 0.05% sodium azide. Peripheral Blood (PB) and BM samples were previously lysed in ammonium chloride lysis solution (0.155 mM NH_4_Cl, 0.01 mM KHCO_3_, 10^−4^ mM EDTA).

The human (h) CD18 antibody used was the 6.7 clone (#551060, BD Bioscience). For the study of the very primitive phenotype of the HSCs, hCD34 (#25-0349-41, eBioscience, San Diego, CA, USA), hCD38 (#302522, Biolegend, San Diego, CA, USA), hCD90 (#555596, BD), and hCD45RA (#47–0458, eBioscience) markers were used and hCD18 (#551060, BD Biosciences) was added as an additional label. Gating strategy used for the analyses of progenitors is included in Fig. S1. Analyses of integrins CD18 and CD49f were performed with clones 6.7 (#551060, BD Biosciences) and GoH3 (#563271, BD Biosciences), respectively.

For cell sorting, freshly isolated CD34^+^ cells obtained from cord blood, as has previously been described, were stained with hCD18 (#551060, BD Bioscience) and hCD34 (#345802, BD Bioscience) monoclonal antibodies and were FACS-sorted using BD INFLUX (BD Biosciences) to separate CD34^+^CD18^high^ and CD34^+^CD18^low/neg^ populations. Cell sorting strategy is included in Fig. S4.

### Clonogenic studies of hematopoietic progenitor cells

CFCs assays were performed in a methylcellulose-based media (#130-091-280 MACS, Miltenyi Biotec). Triplicates of 250 cells from each condition (CD34^+^CD18^high^ and CD34^+^CD18^low/neg^ sorted cells or CD34^+^ total population) were cultured for 24 h, were plated in 35 mm dishes (#430165, Corning, New York, USA), and were grown at 37 °C in 5%CO_2_. Fourteen days after culture, colonies were observed by inverted microscope (Nikon Phase Contrast ELWD 0.3133909, Japan) to identify the different hematopoietic precursors: colony-forming unit granulocyte-macrophage (CFU-GM), burst-forming unit erythroid (BFU-E), and colony-forming unit granulocyte, erythroid, macrophage, megakaryocyte (CFU-GEMM).

### Cell cycle analyses

For the cell cycle analysis, 1.5 × 10^6^ purified CD34^+^ cells from cord blood were suspended in Hoeschst Binding Buffer (Hank’s Balanced Salt Solution, containing 20 mM HEPES, 5.5 mM glucose and 10% HyClone) and stained for DNA and RNA with Hoeschst 33342 (Sigma-Aldrich, St. Louis, Missouri, USA) for 45 min at 37 °C in darkness. Then, pyronin Y (#P9172-IG, Sigma-Aldrich) was added and cells were incubated in the same conditions. Finally, cells were surfaced stained with hCD34 (#555824, BD), hCD38 (#303522, BioLegend), and hCD18 (#555923, BD Pharmingen) monoclonal antibodies and were analyzed by flow cytometry.

### Animals

NOD-scid IL2Rg^null^ (NSG) mice were obtained from the Jackson Laboratory (Bar Harbor, ME) and maintained in micro-isolators. The experimental procedures involving mice were carried out at the CIEMAT animal facility (registration number 28079-21 A). Mice were maintained under high standard conditions (high-efficiency particulate-filtered air, regulated temperature of 22 °C, light/dark cycle of 12 h, and food and ultraviolet-irradiated water ad libitum) and routinely screened for pathogens.

All experimental procedures were carried out according to Spanish and European regulations (Spanish RD 53/2013 and Law 6/2013 that translate and comply with the European Directive 2010/63/UE about the use and protection of vertebrate mammals used for experimentation and other scientific purposes).

### Xenotansplants in NSG mice

NSG mice were irradiated with 1.5 Gy 24 h prior transplantation. Approximately 10^5^ CD34^+^ cells were transplanted into NSG mice by retro-orbital injection. Once a month, bone marrow aspirations were performed to analyze in BM cells the engraftment and lineage distribution by FACS, using hCD45 (#304014, BioLegend), hCD19 (#25-0198, eBioscience), hCD33 (#A07775, Beckman Coulter), hCD34 (#555824, Becton Dickinson), and CD18 (#555923 or #551060, BD Pharmingen) monoclonal antibodies. Transplanted mice were culled at 3 months post-transplantation (mpt) and total bone marrow cells (BMCs) were also analyzed by FACS with the same antibodies. Human CD45^+^ magnetic-labeled cells were selected using CD45 MicroBead Kit (MACS, Miltenyi Biotec, Bergisch Gladbach, Germany) and secondary transplants in NSG mice were performed with comparable number of cells in all groups (3–5 × 10^6^ cells per mouse). After 3 months follow-up, secondary recipients were sacrificed by cervical dislocation and BM and PB samples were analyzed by flow cytometry.

### Limiting dilution assay

CD34^+^ from cord blood samples were sorted out to purify CD34^+^CD18^high^ and CD34^+^CD18^low/neg^ populations as described in Fig. S4. These cells were transplanted into 1.5 Gy irradiated NSG mice at different cell doses (15,000, 2000, and 200 cells/mouse) by retro-orbital injection. Ten months after transplantation, animals were sacrificed by cervical dislocation and BM cells were harvested and analyzed by flow cytometry for the presence of human hematopoietic cells, as well as for myeloid and B cells using hCD45^+^ (#A07782, Beckman Coulter, Brea, CA, USA), hCD33 (#A07775, Beckman Coulter), and hCD19 (#363012, BioLegend) monoclonal antibodies. Results were analyzed using the ELDA software, as described by Hu and Smyth [9].

### Statistical analyses

The statistical analysis was performed using GraphPad Prism version 7.00 for Windows (GraphPad Software, San Diego, USA, www.graphpad.com). For comparison of qualitative and quantitative variables in which the sample size (*n*) < 30 or data did not follow a normal distribution, deduced from Kolmogorov-Smirnov test, Kruskal-Wallis test was performed to elucidate the significance of differences in the whole group. If samples showed significant differences, established when *P* < 0.05, a nonparametric two-tailed Mann-Whitney test was performed to obtain the *P* value or the adjusted *P* value when Dunn’s multiple comparison test was applied. The significances are expressed as *P* < 0.0001 (****), *P* < 0.001 (***), *P* < 0.01 (**), or *P* < 0.05 (*).

## Results

### The low/negative expression of CD18 defines a primitive phenotype of cord blood CD34^+^ cells

**Fig. 1:**
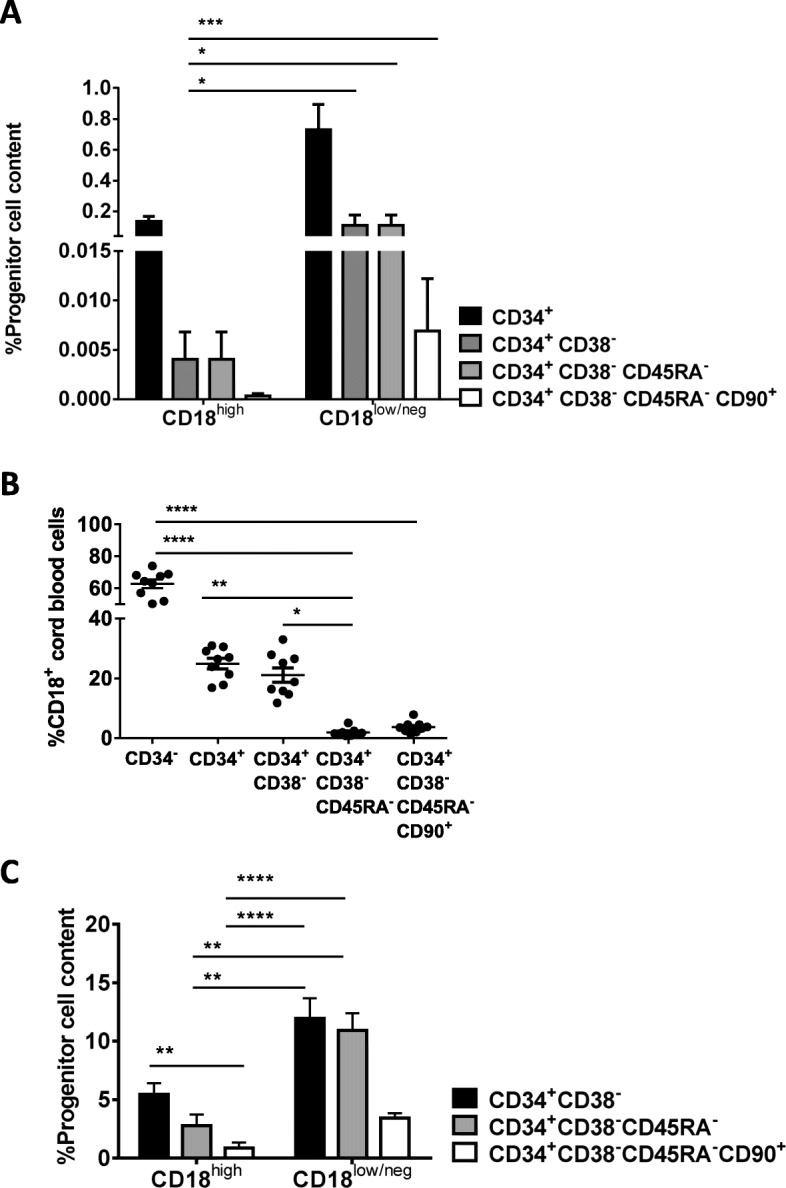
Flow cytometry analyses of CD18 expression in different cord blood HSPCs populations. **a** Analysis of the proportion of CD18^high^ and CD18^low/neg^ cells in different HSPCs present in nucleated cord blood cells. Percentages in the total nucleated cord blood cell population are shown. **b** Percentage of CD18^+^ cells in different HSPCs analyzed in purified CD34^+^ cord blood samples. **c** Percentage of HSPCs in purified CD34^+^ cells expressing high (CD18^high^) or low levels (CD18^low/neg^) of CD18. In panels **b** and **c**, CD34^+^ cells were purified by magnetic sorting prior to conducting flow cytometry studies. The significance of differences between groups is expressed as *P* < 0.0001 (****), *P* < 0.001 (***), *P* < 0.01 (**), or *P* < 0.05 (*)

In a first set of studies, mononuclear CB cells were classified based on their CD18 expression as CD18^high^ and CD18^low/neg^ (gating strategy Fig. S1A). In each of these two populations, the content of CD34^+^, CD34^+^CD38^−^, CD34^+^CD38^−^CD45RA^−^, and CD34^+^CD38^−^CD45RA^−^CD90^+^ was analyzed. These analyses revealed that the proportion of CD34^+^ cells was significantly higher (5-fold) in the CD18^low/neg^ fraction as compared to CD18^high^ cells. Also, evident increases in primitive populations were observed in the CD18^low/neg^ fraction when other HSC markers were considered. Significantly, the proportion of very primitive CD34^+^CD38^−^CD45RA^−^CD90^+^ HSCs was 23 times higher in the CD18^low/neg^ population as compared to the CD18^high^ population (7 × 10^−3^% vs 4 × 10^−4^%, respectively) (Fig. 1a), confirming that the reduced expression of CD18 was associated with an increased proportion in cells with a primitive HSC phenotype. Thereafter, the proportion of CD18^+^ cells was investigated in CD34^+^-enriched HSCs according to CD34, CD38, CD45RA, and CD90 markers (gating strategy Fig. S1B). In contrast to data obtained in CD34^−^ cells, where 65% of these cells were CD18^+^, only 25% of the CD34^+^ or CD34^+^CD38^−^ cells were CD18^+^. Moreover, this percentage was markedly decreased when CD34^+^ cells with a more primitive phenotype (CD34^+^CD38^−^CD45RA^−^ or CD34^+^CD38^−^CD45RA^−^CD90^+^ cells) were analyzed (Fig. 1b). Similar studies were conducted with BM and mPB cells. As observed in CB samples, in both cases, the proportion of CD18^+^ cells was decreased in populations defined by a primitive HSC phenotype (Fig. S2). Consistent with the above observations, when CD34^+^ cells were sorted, analyses of CD18^high^ and CD18^low/neg^ populations confirmed a higher proportion of cells with a primitive HSC phenotype observed in all instances in CD18^low/neg^ cells as compared to CD18^high^ cells (Fig. 1c).

Recently published papers have shown the close relationship between different integrin’s expression and the primitiveness of HSCs. Particularly, the expression of integrin α_6_ (CD49f) in HSCs has been correlated with long-term multi-lineage repopulation ability in NSG mice. Thus, we have compared the expression levels of CD49f with CD18 in CD34^+^ and CD34^+^CD38^−^ populations [10]. As observed in Fig. S3, no co-expression of CD18 was observed in CD49f^+^ cells in these HSPC populations.

**Fig. 2:**
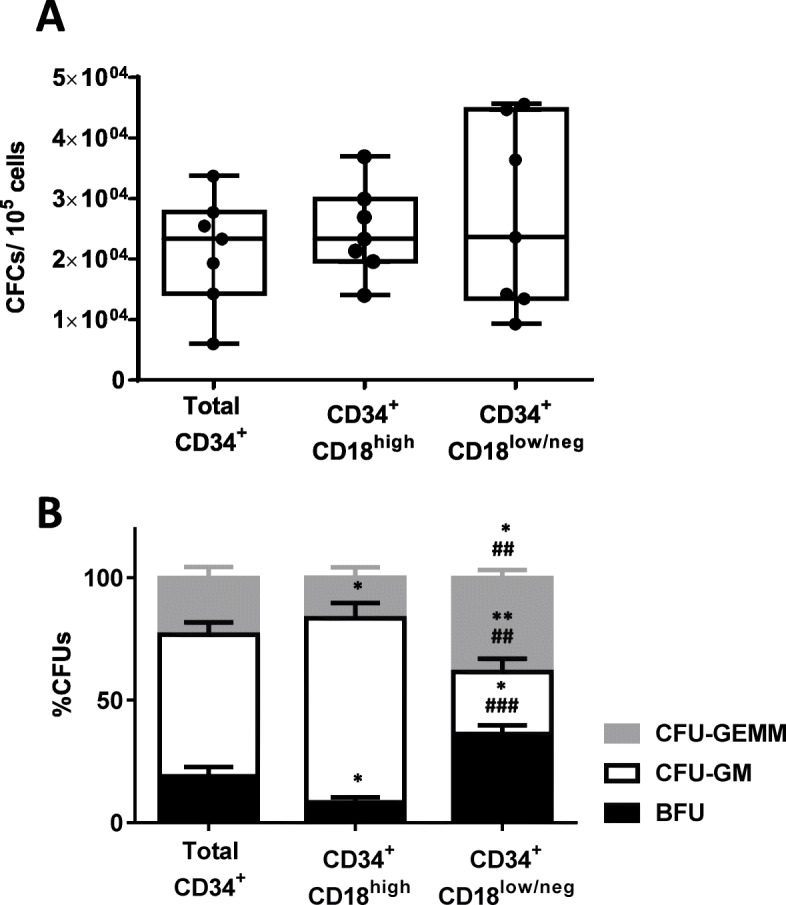
Assessment of the colony-forming cell content in different cord blood CD18 expressing populations. **a** Analysis of the content of total CFCs per 10^5^ seeded cells and **b** distribution of the different types of CFCs in CD34^+^ cells sorted on the basis of CD18 expression. CFU-GM, BFU-E, and CFU-GEMM are shown. The significance of differences between total and CD18^high^ or CD18^low/neg^ sorted populations is shown as *P* < 0.01(**) or *P* < 0.05(*). The significance of differences between CD18^high^ and CD18^low/neg^ populations is shown as *P* < 0.001 (^###^) or *P* < 0.01 (^##^)

**Fig. 3:**
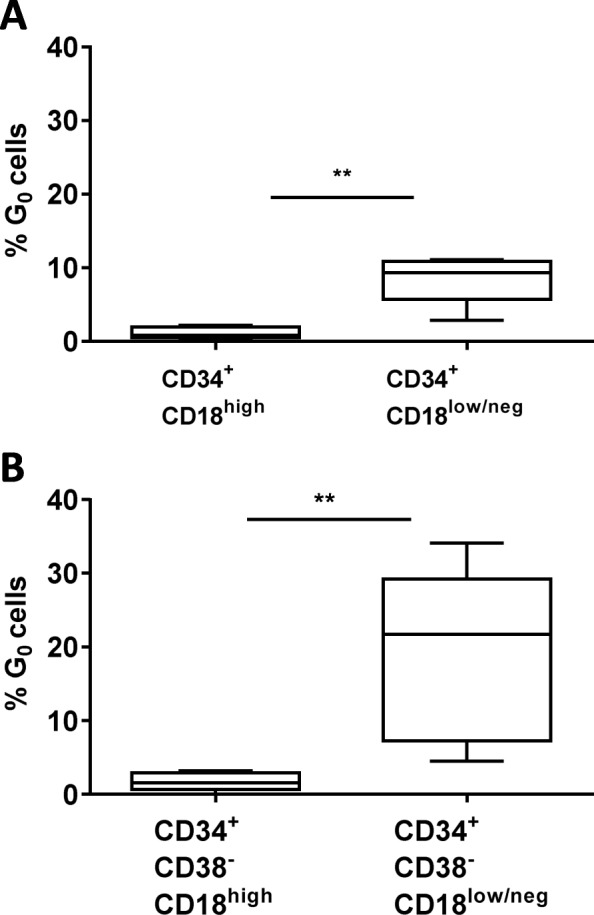
Cell cycle analyses in different cord blood CD18 expressing populations. **a** Cell cycle analyses in CD34^+^CD18^high^ and CD34^+^CD18^low/neg^ cells. **b** Cell cycle analyses in CD34^+^CD38^−^CD18^high^ and CD34^+^CD38^−^CD18^low/neg^ cells. Graphics show the percentage of cells in G_0_ phase. The significance of differences between groups is shown as *P* < 0.01 (**)

In subsequent studies, the content of CFCs was assessed in purified CB CD34^+^ cells and in CD34^+^CD18^high^ and CD34^+^CD18^low/neg^ populations (see gating strategy in Fig. S4). Figure 2 shows the results in which seven CB samples were analyzed. While no significant differences were observed in the total number of colonies generated from these three different cell populations (Fig. 2a), significant changes were observed in the distribution of the different types of colonies. Whereas the proportion of BFU-E and CFU-GEMM colonies was significantly higher in CD34^+^CD18^low/neg^ cells, this fraction contained a significantly lower number of CFU-GM colonies as compared to the other two ones (Fig. 2b). Since a characteristic primitive HSC phenotype is their resting state, in the next set of experiments, we investigated the percentage of cells in the G_0_ phase in purified CD34^+^CD18^low/neg^ and CD34^+^CD18^high^ cells. As shown in Fig. 3a, a significant increase in the proportion of cells in G_0_ was observed in CD34^+^CD18^low/neg^ as compared to CD34^+^CD18^high^ cells (8.5% vs 1.2%, respectively). A higher proportion of cells in G_0_ was even observed when primitive CD34^+^CD38^−^CD18^low/neg^ cells were analyzed (19%), while only 1.8% of CD34^+^CD38^−^CD18^high^ cells were in G_0_.

Taken together, the results of these in vitro studies indicate that the low membrane expression of CD18 in purified HSPCs endows a population with a high content in erythroid and mixed CFCs and with a high content in quiescent cells.

### The low/negative expression of CD18 defines HSPCs with extended repopulating ability

In an attempt to quantify the HSC content in CD34^+^CD18^high^ and CD34^+^CD18^low/neg^ cells, a limiting dilution assay was performed. A total of 44 mice were transplanted with different numbers of purified CD34^+^CD18^high^ or CD34^+^CD18^low/neg^ CB cells. The evaluation of the human hematopoietic repopulation was always assessed at 2.5 months post-transplantation (Fig. S5A). Animals were considered positive when at least 0.1% hCD45^+^, hCD33^+^, and hCD19^+^ cells were determined in recipient BM. Data from the Extreme Limiting Dilution Analysis (ELDA; Fig. S5B) revealed that no significant differences in the content of short-term hematopoietic repopulating cells were observed between the CD34^+^CD18^high^ and CD34^+^CD18^low/neg^ cell populations.

**Fig. 4:**
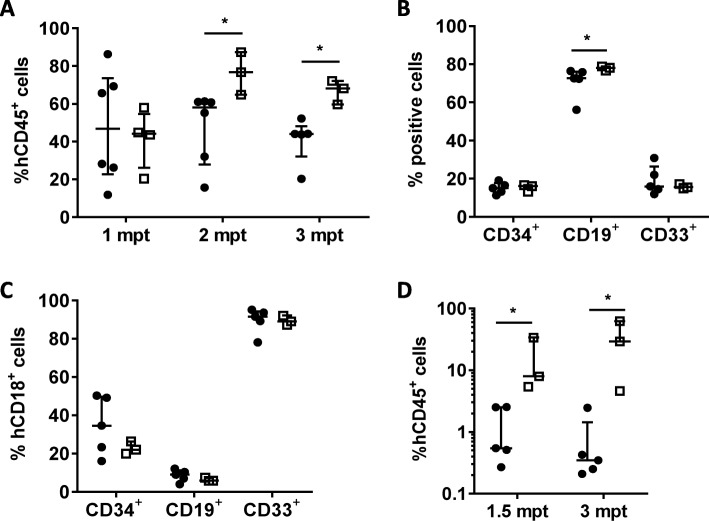
In vivo repopulating potential of cord blood CD34^+^ cells expressing different levels of CD18. **a** hCD45^+^ levels at different months post-transplant in the BM of recipient mice transplanted with purified CD34^+^CD18^high^ (*n* = 6; black circles) or CD34^+^CD18^low/neg^ (*n* = 4; open squares) populations. Graphic represents the percentage of positive cells in total BM. **b** Lineage distribution represented as the percentage of human CD34^+^, CD19^+^, and CD33^+^ and **c** the percentage of CD18^*+*^ cells in these populations determined in mouse BM at 3 mpt. **d** hCD45^+^ levels at 3 mpt in the BM of secondary recipients that were transplanted with 3–5 × 10^6^ purified hCD45^+^ cells obtained from primary recipients at 3 mpt (CD34^+^CD18^high^, *n* = 5; CD34^+^CD18^low/neg^*, n* = 3). Graphic represents the percentage of positive cells in total BM. The significance of differences between groups is expressed as *P* < 0.05 (*)

Due to this striking observation, we investigated potential differences in the stemness of CD34^+^CD18^high^ and CD34^+^CD18^low/neg^ cells by conducting additional repopulation studies in NSG mice. In these studies, 100,000 CD34^+^CD18^low/neg^ or CD34^+^CD18^high^ cells were transplanted into NSG mice. Levels of engraftment were then analyzed at different times post-transplantation and also after re-transplantation into secondary recipients. Although a similar proportion of BM hCD45^+^ cells was observed in analyses conducted at 1 mpt, higher levels of hCD45^+^ cells were observed at 2 and 3 mpt in mice transplanted with CD34^+^CD18^low/neg^ cells compared to the CD34^+^CD18^high^ group (Fig. 4a). In both groups, similar distributions of hCD34^+^, hCD19^+^, and hCD33^+^ populations were observed in hCD45^+^ engrafted cells (Fig. 4b). Neither differences in the proportion of CD18^+^ cells were found in hCD34^+^, hCD19^+^, and hCD33^+^ cells generated in mice transplanted with CD34^+^CD18^low/neg^ or CD34^+^CD18^high^ cells (Fig. 4c), revealing the re-expression of CD18 in hematopoietic cells generated from the primitive CD34^+^CD18^low/neg^ repopulating cells.

At 3 mpt, primary recipients from each of the two recipient groups were sacrificed and BM cells were pooled and hCD45^+^ cells purified by magnetic cell sorting. The same number of hCD45^+^ cells was then transplanted into secondary NSG recipients to evaluate the long-term repopulating ability of CD34^+^CD18^high^ and CD34^+^CD18^low/neg^ cells that had been transplanted into primary recipients. Remarkably, the proportion of CD45^+^ cells in the BM of secondary recipients was around 10-fold higher in secondary recipients corresponding to the CD34^+^CD18^low/neg^ group (Fig. 4d), confirming the enhanced long-term repopulation ability of CD34^+^CD18^low/neg^ cells as compared to CD34^+^CD18^high^ cells.

## Discussion

Due to the difficulties in the identification of a unique marker characteristic of primitive HSCs, several marker combinations have been proposed, which have markedly improved our knowledge about the functional properties of HSCs, and also enabled the classification and sorting of these cells for different biological and clinical applications. Although the CD34^+^ marker is the most frequently used in clinical practice, there is a strong consensus that Lin^−^CD34^+^CD38^−^CD45RA^−^CD90^+^ cells constitute a highly purified population of self-renewing HSCs [11, 12].

Among the other markers that have been used for the characterization of the HSCs, the membrane expression of certain β_1_ integrins has been observed in very primitive HSC subsets and has shown the functional role of these integrins in HSCs. This was the case of CD49b (integrin α_2_), whose expression in CD34^+^CD38^−^ cells and also in the more primitive CD34^+^CD38^−^CD90^+^ population correlated with the long-term repopulating ability of these cells in NSG mice [13]. Likewise, expression of CD49f (integrin α_6_) has been observed in HSC subsets with long-term multi-lineage repopulation ability in NSG mice. Thus, the very primitive HSCs have been defined as a Lin^−^CD34^+^CD38^−^CD45RA^−^CD90^+^CD49f^+^ population, this last marker being absent in more differentiated multipotent progenitor cells [10]. Additionally, the expression of CD49d (integrin α_4_) has also been associated with the primitiveness of the HSCs and involved in the homing of adult HSCs in BM [14].

Although previous studies have shown the role of certain integrins in the interaction of HSCs with other cells in the BM niche [6, 15–17], in the case of β_2_ integrins, previous studies have revealed the lack of expression of both CD11A and CD11B subunits in primitive HSCs [4–8]. Additionally, no studies have been performed to elucidate the implication of CD18 expression in the HSPC phenotype.

Our first set of studies showed that CD18^low/neg^ cells contain a higher proportion of primitive HSCs defined by the following expression markers: CD34^+^CD38^−^CD45RA^−^CD90^+^. Functionally, CD18^low/neg^ cells were enriched in CFU-GEMM, more primitive than the CFU-GM progenitors. Additionally, a higher content of cells in G_0_ was observed in primitive progenitors with a low/negative CD18 expression, consistent with the quiescent nature of HSCs in healthy donors.

Limiting dilution assays performed to quantify the enrichment of short-term repopulating HSPCs in the different CD34^+^ cell subpopulation did not show significant differences between CD18^low/neg^ and CD18^high^ cells. Nevertheless, when secondary transplantation studies were conducted, marked differences in levels of hematopoietic reconstitution were observed between CD18^low/neg^ and CD18^high^ cells, strongly suggesting that the CD18^low/neg^ cell fraction defines a more primitive population of long-term repopulating cells.

These observations thus indicate that CD18 expression in the cell membrane of CD34^+^ does not discriminate the short-term repopulating properties of human HSPCs. Nevertheless, the low expression of CD18 in CD34^+^ cells defines primitive HSPCs with extensive repopulating properties. However, the formal demonstration that a very primitive stem cell population is enriched in CD34^+^CD18^low/neg^ cells would require performing very challenging limiting dilution assays in secondary recipients. The subsequent differentiation of these primitive HSPCs result in the upregulated membrane expression of this marker, probably due to changes in their interaction with stromal cells in the HSC niche, as previously proposed [18].

## Conclusion

Taken together, our results show that the low/negative expression of CD18 constitutes a reliable marker which defines a subpopulation of primitive CD34^+^ cells with extended long-term Losada for their collaboration in the administrative work. The authors would also like to thankin vivo repopulating ability.

## Supplementary information

**Additional file 1: Figure S1.** Schematic representation of the gating strategy. (A) Gating strategy for Fig. 1A and C. Nucleated cord blood cells were labeled using hCD18, hCD34, hCD38, hCD90 and hCD45RA. The gating strategy started with the selection of CD18^high^ and CD18^low/neg^ populations. Inside each of these groups of cells, CD34^+^ population was selected. Then, the CD38^-^ population was chosen inside the CD18^high^CD34^+^ and CD18^low/neg^CD34^+^ fractions. These two groups (CD18^high^CD34^+^CD38^-^ and CD18^low/neg^CD34^+^CD38^-^) were asked for the percentage of both CD90^+^ and CD45RA^-^ populations. (B) Gating strategy for Figure 1B. The percentage of CD18^high^ and CD18^low/neg^ populations was determined in the CD34^+^ population. A similar analysis was performed in the CD38^-^ population gated inside the CD34^+^. The CD90^+^CD45RA^-^ population selected inside these CD38^-^ cells was also asked for the percentage of CD18^high^ and CD18^low/neg^ populations.

**Additional file 2: Figure S2.** Flow cytometry analyses of CD18 expression in CD34^-^, CD34^+^ and CD34^+^CD38^-^ cells from BM and mPB. Percentage of CD18^+^ cells in different HSPCs from BM (A) or mPB (B). The significance of differences between groups is expressed as *P*<0.05(*).

**Additional file 3: Figure S3.** Flow cytometry analyses of integrins CD49f and CD18 in purified CD34^+^ cells from cord blood samples. Histograms represent data from three independent CB samples analyzed for the percentage of CD18 and CD49f expressing cells in two different subsets of progenitor populations: CD34^+^ and CD34^+^CD38^-^.

**Additional file 4: Figure S4.** Gating strategy for the cell sorting of CD34^+^ cells based on the expression of CD18. Histograms represent data from three independent CB samples sorted out based on CD34 and CD18 expression.

**Additional file 5: Figure S5.** Limiting dilution assay deduced from the transplantation of CD34^+^CD18^high^ and CD34^+^CD18^low/neg^ CB cells. Limiting dilution assay performed with CD34^+^CD18^high^ and CD34^+^CD18^low/neg^ CB cells transplanted into NSG mice. Purified CD34^+^CD18^high^ and CD34^+^CD18^low/neg^ cells from three different pools of CB samples were transplanted into immunodeficient NSG mice. Ten weeks after the infusion (2.5 mpt), BM cells were harvested and analyzed by flow cytometry for the presence of hCD45^+^ cells. (A) Table summarizing the number of engrafted mice (mice with ≥ 0.1% hCD45^+^ cells and ≥ 0.1% hCD33^+^ and ≥ hCD19^+^ cells) per total number of transplanted mice in each cell dose, as well as the stem cell frequency in a 95% confidence interval. Analyses were performed using the ELDA software [9]. (B) Log-fraction plot of the limiting dilution model. Slope of solid lines shows the active cell fraction. Dotted lines represent the 95% confidence interval. Triangles symbolize groups with no negative response (CD34^+^CD18^high^ and CD34^+^CD18^low/neg^ at 15,000 cell dose).

## Data Availability

Data sharing not applicable to this article as no datasets were generated or analyzed during the current study.

## Abbreviations

APC: Antigen-presenting cell
BFU-E: Burst-forming unit erythroid
BM: Bone marrow
CB: Cord blood
CFC: Colony-forming cell
CFU-GEMM: Colony-forming unit granulocyte, erythroid, macrophage, megakaryocyte
CFU-GM: Colony-forming unit granulocyte-macrophage
ELDA: Extreme limiting dilution assay
FACS: Flow Cytometry Activated Cell Sorting
HSCs: Hematopoietic stem cells
HSPC: Hematopoietic stem and progenitor cell
IRB: Institutional Review Board
LFA-1: Lymphocyte function-associated antigen 1
Mac-1: Macrophage 1 antigen
mPB: Mobilized peripheral blood
PB: Peripheral blood
P/S: Penicillin/streptomycin

## References

[1] Hynes RO (2002). Integrins: bidirectional, allosteric signaling machines. Cell.

[2] Zhang Y, Wang H (2012). Integrin signalling and function in immune cells. Immunology.

[3] Tan SM (2012). The leucocyte beta2 (CD18) integrins: the structure, functional regulation and signalling properties. Biosci Rep.

[4] Torensma R (1996). Induction of LFA-1 on pluripotent CD34+ bone marrow cells does not affect lineage commitment. Blood.

[5] Torensma R (2000). Regulation of LFA-1 expression by CD34 positive cells and inducible growth factor production by stroma enable formation of bone marrow compartments; Subject Heading. Hematology.

[6] Gunji Y (1992). Expression and function of adhesion molecules on human hematopoietic stem cells: CD34+ LFA-1- cells are more primitive than CD34+ LFA-1+ cells. Blood.

[7] Fathman JW (2014). Upregulation of CD11A on hematopoietic stem cells denotes the loss of long-term reconstitution potential. Stem Cell Reports.

[8] Van Beusechem VW (1994). Gene transfer into nonhuman primate CD34+CD11b- bone marrow progenitor cells capable of repopulating lymphoid and myeloid lineages. Hum Gene Ther.

[9] Hu Y, Smyth GK (2009). ELDA: extreme limiting dilution analysis for comparing depleted and enriched populations in stem cell and other assays. J Immunol Methods.

[10] Notta F (2011). Isolation of single human hematopoietic stem cells capable of long-term multilineage engraftment. Science.

[11] Majeti R, Park CY, Weissman IL (2007). Identification of a hierarchy of multipotent hematopoietic progenitors in human cord blood. Cell Stem Cell.

[12] Boulais PE, Frenette PS (2015). Making sense of hematopoietic stem cell niches. Blood.

[13] Wong WM (2013). Expression of integrin alpha2 receptor in human cord blood CD34+CD38-CD90+ stem cells engrafting long-term in NOD/SCID-IL2Rgamma(c) null mice. Stem Cells.

[14] Scott LM, Priestley GV, Papayannopoulou T (2003). Deletion of alpha4 integrins from adult hematopoietic cells reveals roles in homeostasis, regeneration, and homing. Mol Cell Biol.

[15] Gigant C (2003). CD34+ cells homing: quantitative expression of adhesion molecules and adhesion of CD34+ cells to endothelial cells exposed to shear stress. Biorheology.

[16] Gigant C (2001). Quantitative expression of adhesion molecules on granulocyte colony-stimulating factor-mobilized peripheral blood, bone marrow, and cord blood CD34+ cells. J Hematother Stem Cell Res.

[17] Mohle R (1995). Differential expression of L-selectin, VLA-4, and LFA-1 on CD34+ progenitor cells from bone marrow and peripheral blood during G-CSF-enhanced recovery. Exp Hematol.

[18] Gomez JC, Doerschuk CM (2010). The role of CD18 in the production and release of neutrophils from the bone marrow. Lab Investig.

