# Supplementary figures and images for: The downregulated membrane expression of CD18 in CD34+ cells defines a primitive population of human hematopoietic stem cells
Source: Stem Cell Res Ther. 2020 Apr 28;11:164. doi: 10.1186/s13287-020-01672-0 (PMC7189462; doi:10.1186/s13287-020-01672-0)

Figure S1

A

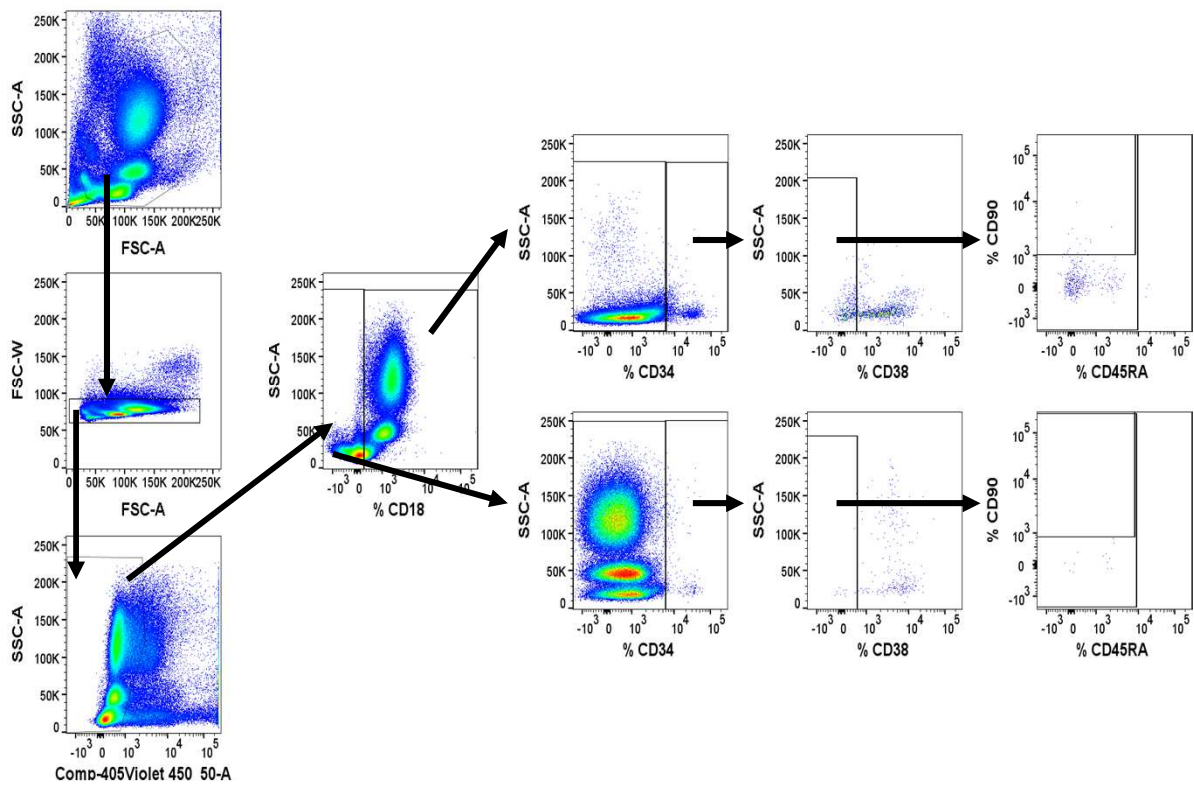

B

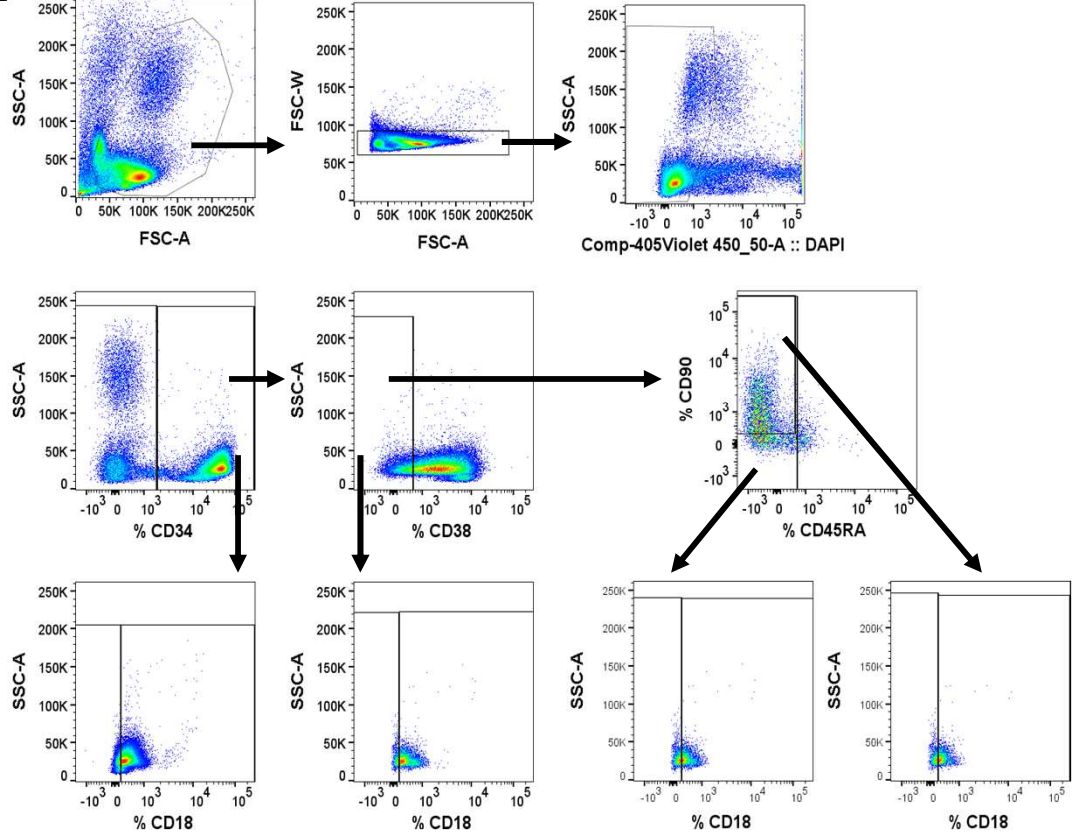

Supplement: Supplementary file 1 — Additional file 1: Figure S1. Schematic representation of the gating strategy. (A) Gating strategy for Fig. 1A and C. Nucleated cord blood cells were labeled using hCD18, hCD34, hCD38, hCD90 and hCD45RA. The gating strategy started with the selection of CD18high and CD18low/neg populations. Inside each of these groups of cells, CD34+ population was selected. Then, the CD38- population was chosen inside the CD18highCD34+ and CD18low/negCD34+ fractions. These two groups (CD18highCD34+CD38- and CD18low/negCD34+CD38-) were asked for the percentage of both CD90+ and CD45RA- populations. (B) Gating strategy for Figure 1B. The percentage of CD18high and CD18low/neg populations was determined in the CD34+ population. A similar analysis was performed in the CD38- population gated inside the CD34+. The CD90+CD45RA- population selected inside these CD38- cells was also asked for the percentage of CD18high and CD18low/neg populations. [file 13287_2020_1672_MOESM1_ESM.pdf]

Figure S2

A

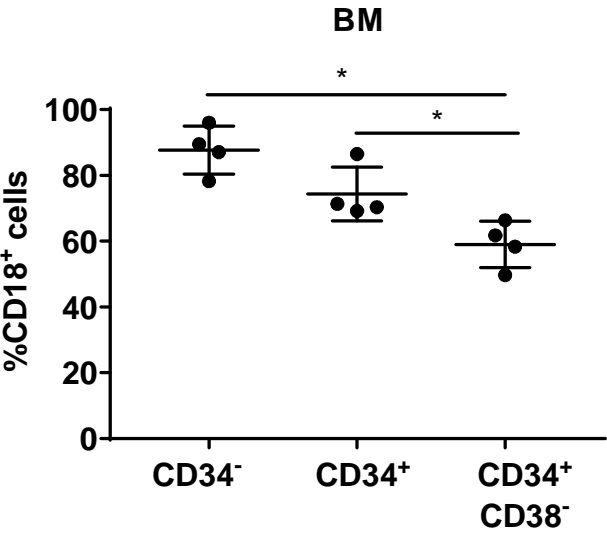

B

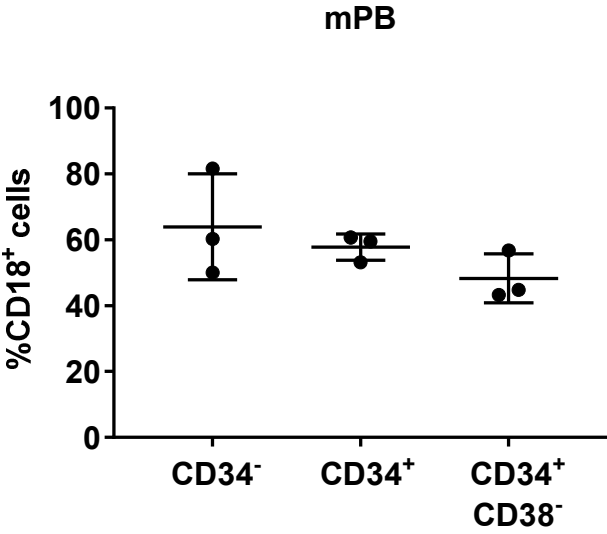

Supplement: Supplementary file 2 — Additional file 2: Figure S2. Flow cytometry analyses of CD18 expression in CD34-, CD34+ and CD34+CD38- cells from BM and mPB. Percentage of CD18+ cells in different HSPCs from BM (A) or mPB (B). The significance of differences between groups is expressed as P<0.05(*). [file 13287_2020_1672_MOESM2_ESM.pdf]

Figure S3

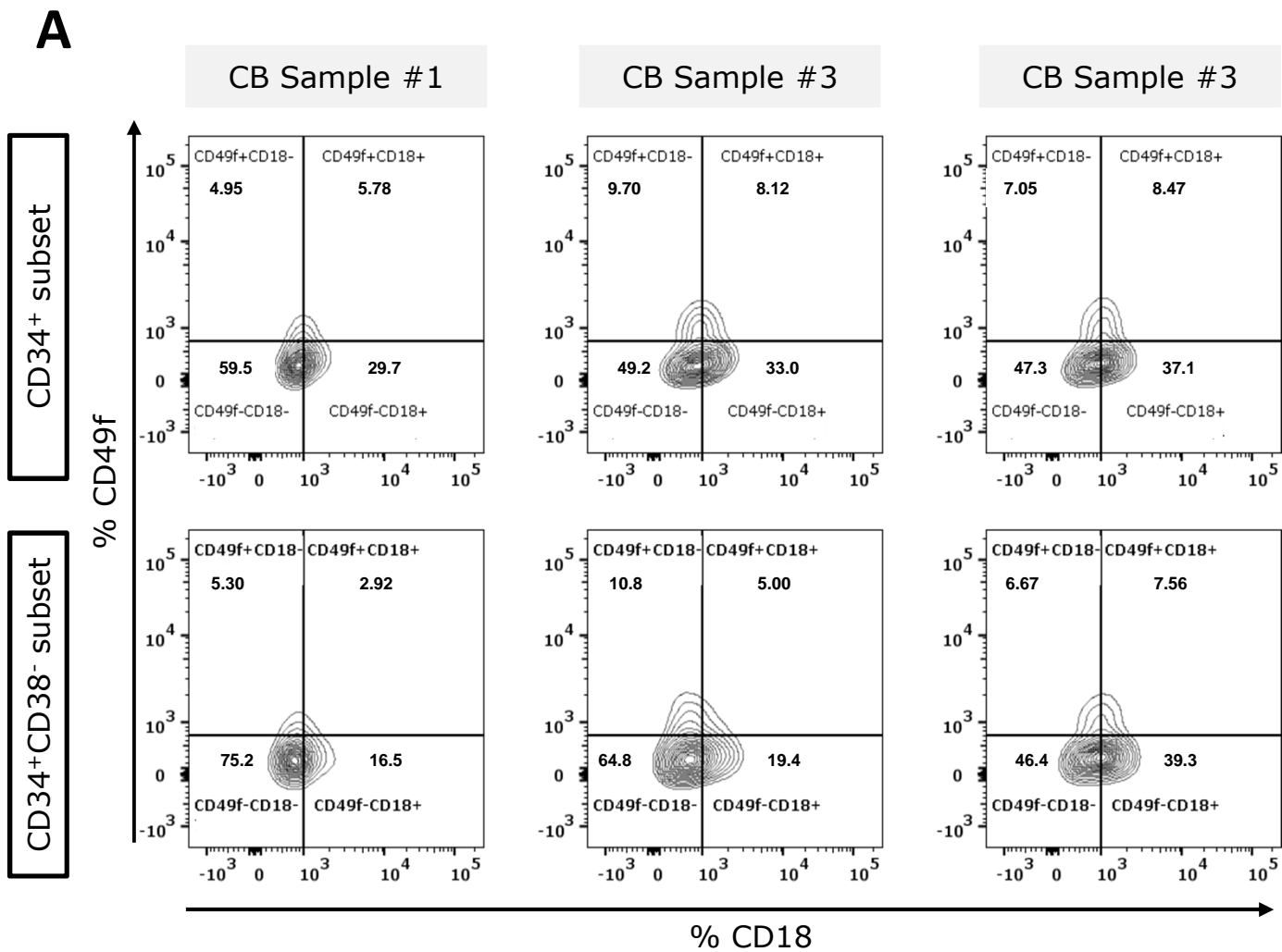

Supplement: Supplementary file 3 — Additional file 3: Figure S3. Flow cytometry analyses of integrins CD49f and CD18 in purified CD34+ cells from cord blood samples. Histograms represent data from three independent CB samples analyzed for the percentage of CD18 and CD49f expressing cells in two different subsets of progenitor populations: CD34+ and CD34+CD38-. [file 13287_2020_1672_MOESM3_ESM.pdf]

Figure S4

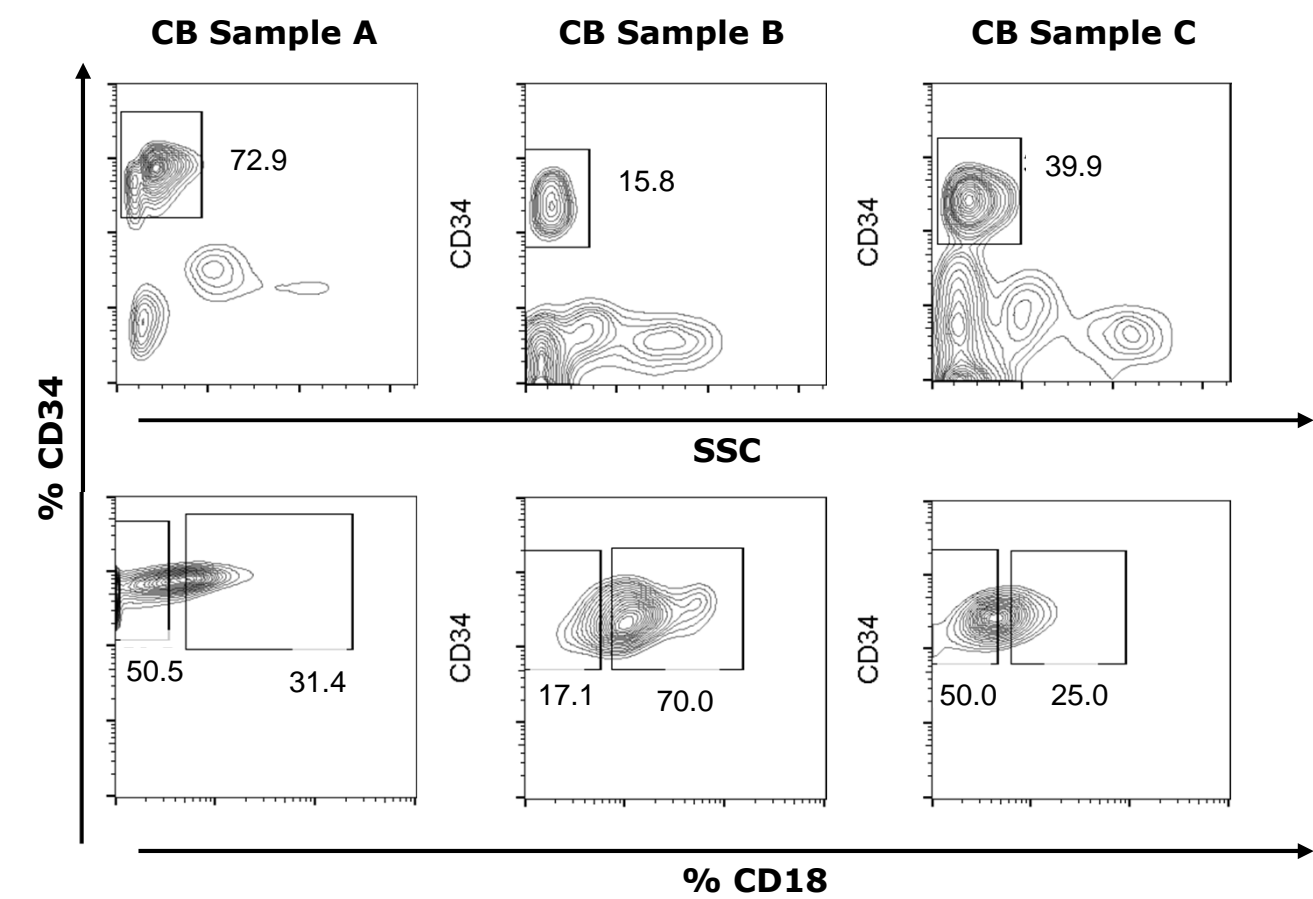

Supplement: Supplementary file 4 — Additional file 4: Figure S4. Gating strategy for the cell sorting of CD34+ cells based on the expression of CD18. Histograms represent data from three independent CB samples sorted out based on CD34 and CD18 expression. [file 13287_2020_1672_MOESM4_ESM.pdf]
