# Supplementary material for: The downregulated membrane expression of CD18 in CD34+ cells defines a primitive population of human hematopoietic stem cells
Source: Stem Cell Res Ther. 2020 Apr 28;11:164. doi: 10.1186/s13287-020-01672-0 (PMC7189462; doi:10.1186/s13287-020-01672-0)

Figure S5

A

Transplanted cell number	CD18^{high}	CD18$^{low/neg}$
**15,000**	5/5	6/6
**2,000**	4/9	5/10
**200**	0/7	0/7
**Stem cell frequency**	3496	3041
**95% confidence interval range (upper-lower)**	1514-8072	1380-6700
p value	0.817	

B

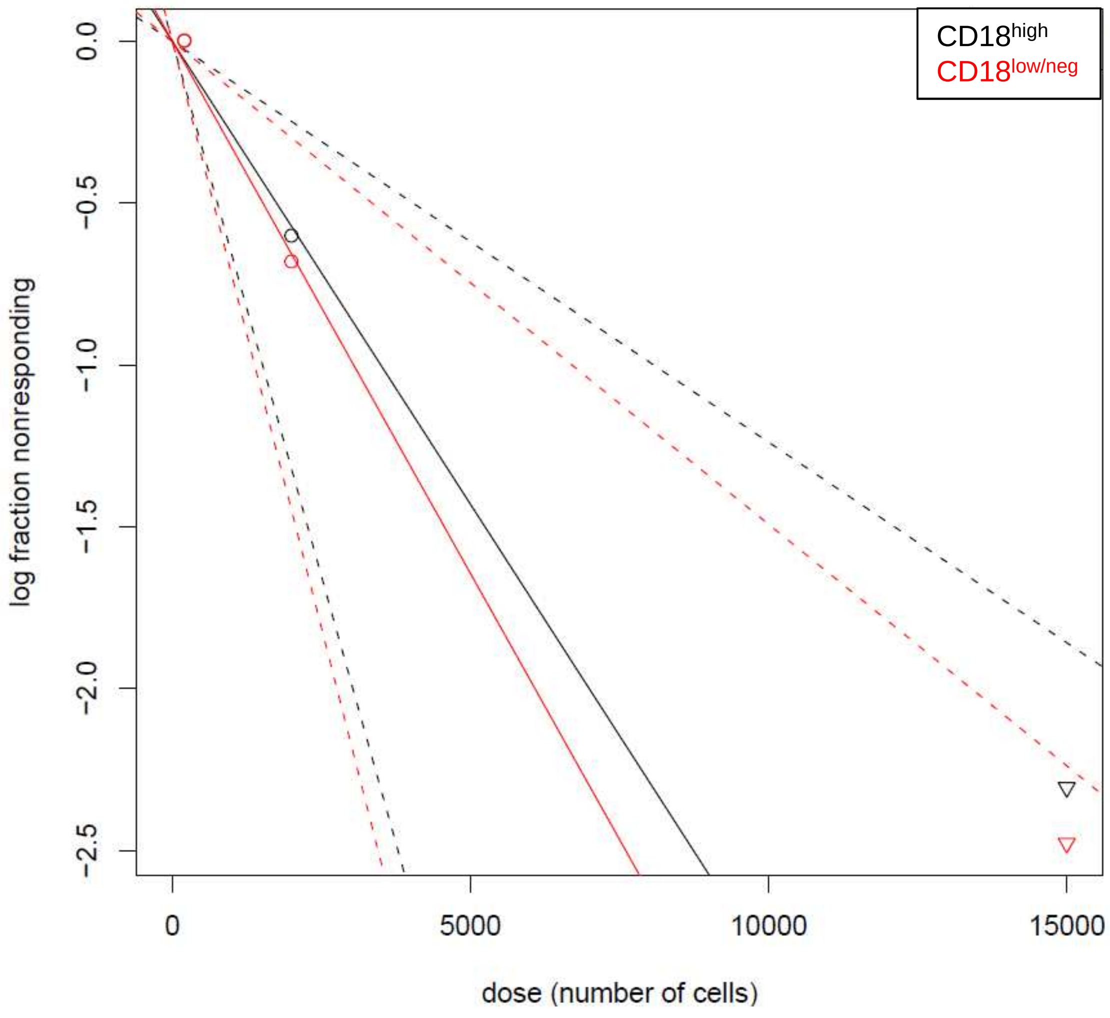

Supplement: Supplementary file 5 — Additional file 5: Figure S5. Limiting dilution assay deduced from the transplantation of CD34+CD18high and CD34+CD18low/neg CB cells. Limiting dilution assay performed with CD34+CD18high and CD34+CD18low/neg CB cells transplanted into NSG mice. Purified CD34+CD18high and CD34+CD18low/neg cells from three different pools of CB samples were transplanted into immunodeficient NSG mice. Ten weeks after the infusion (2.5 mpt), BM cells were harvested and analyzed by flow cytometry for the presence of hCD45+ cells. (A) Table summarizing the number of engrafted mice (mice with ≥ 0.1% hCD45+ cells and ≥ 0.1% hCD33+ and ≥ hCD19+ cells) per total number of transplanted mice in each cell dose, as well as the stem cell frequency in a 95% confidence interval. Analyses were performed using the ELDA software [9]. (B) Log-fraction plot of the limiting dilution model. Slope of solid lines shows the active cell fraction. Dotted lines represent the 95% confidence interval. Triangles symbolize groups with no negative response (CD34+CD18high and CD34+CD18low/neg at 15,000 cell dose). [file 13287_2020_1672_MOESM5_ESM.pdf]
